# Comparison of transcriptome profiles of radiotherapy beams on MCF-7 breast cancer xenografts

**DOI:** 10.1007/s12282-025-01735-1

**Published:** 2025-06-27

**Authors:** Tuğba Kul Köprülü, Serhat Aras, Burçin Erkal Çam, Altan Kara

**Affiliations:** 1 Experimental Medicine Application and Research Center, University of Health Sciences, Uskudar, Istanbul, Turkey; 2 Department of Molecular Medicine, Hamidiye Instıtute of Health Sciences, University of Health Sciences, Uskudar, Istanbul, Turkey; 3 Department of Radiation Oncology, Haydarpasa Numune Training and Research Hospital, University of Health Sciences, Uskudar, Istanbul, Turkey; 4 Medical Imaging Techniques, Department of Medical Services and Techniques, Hamidiye Vocational School of Health Services, University of Health Sciences, Uskudar, Istanbul, Turkey; 5 Department of Molecular Biology and Genetics, Faculty of Arts and Sciences, Yıldız Technical University, Esenler, Istanbul, Turkey; 6 Genalyse Genetic Analysis and Reporting Ltd., Istanbul, Turkey

**Keywords:** Radiotherapy, Breast cancer, Xenografts, Transcriptome, Next Generation Sequencing

## Abstract

The objective of this study was to investigate the acute radiobiological mechanisms underlying Flattening Filter (FF) and Flattening Filter Free (FFF) radiotherapy-induced inhibition of breast cancer (BCa) by detecting changes in gene expression levels. The study involved thirty-six adult nude mice models that were randomly divided into five groups: control (G1), breast cancer (BCa) (G2), FF-400 (G3), FFF-1120 (G4), and FFF-1820 (G5). The control group had no radiation or treatment, while the BCa group had a cancer model but no radiation. The BCa models were subjected to a single dose of 20 Gy of radiotherapy at varying dose rates: 400, 1120, and 1820 MU/min (G3, G4, and G5, respectively). Twenty days after implantation of the MCF-7 cancer cell line, nude mice were irradiated and sacrificed 48 h later for genetic analysis. A transcriptome library was created by extracting RNA from tissue samples. In the differentially expressed genes (DEG) analysis, a total of 1565 genes were found to be significantly altered in the G2 vs. G1 groups. When radiotherapy groups (G3, G4, and G5) were analyzed, 334 DEGs (231 down, 103 up) were identified in G3 vs G2 data, 167 DEGs (103 down, 64 up) in G4 vs G2 data, and 187 DEGs (116 down, 71 up) in G5 vs G2 data. Gene Ontology molecular function analysis revealed significant differences between groups G3 and G2, with an emphasis on protein binding, glutamate receptor activity, and interleukin-8 receptor activation. Similar features were observed when comparing groups G5 and G2. The analysis showed significant differences in the expression of the key ERAD pathway genes *Ufd1* and *Cav1* between the BCa and radiotherapy groups. It was concluded that the FFF beam significantly alters the processes underlying the ERAD pathway and glutamate receptor activation compared to the FF beam.

**Supplementary Information:**

The online version contains supplementary material available at 10.1007/s12282-025-01735-1.

## Introduction

The prevention and progression of breast cancer (BCa) is of great importance due to its high incidence and mortality [1]. The use of modern radiotherapy techniques is crucial in achieving this goal. Although significant progress has been made in treating BCa with surgery, radiotherapy, and chemotherapy, the long-term prognosis of patients remains unsatisfactory due to various factors [2].

During cancer treatment, such as breast, head, neck, and lung cancer, radiotherapy aims to deliver a controlled and precise lethal dose to the tumor tissue while minimizing the dose to healthy tissues outside the targeted volume [3–5]. Significant progress has been made in the treatment of BCa due to developing technology and treatment techniques, especially in the last decade [6]. Technological advances have led to the increasing popularity of Flattening Filter-Free (FFF) beams in cancer radiotherapy [7, 8]. FFF beams allow up to a fourfold increase in dose rate compared to conventional low-dose-rate beams with a Flattening Filter (FF). Clinical applications of radiotherapy often utilize high dose rates with FFF beams to reduce treatment times, allowing for a quicker completion of the radiotherapy procedure [9]. In recent years, high-dose-rate beams have become increasingly popular in Stereotactic Body Radiation Therapy (SBRT), Stereotactic Radiosurgery (SRS), and Volume-Modulated Arc Therapy (VMAT) techniques. This is due to their ability to significantly reduce patient irradiation times [10].

Although radiotherapy has been known to be effective in cancer treatment for some time, a better understanding of how tumors respond to radiation is still required. Next Generation Sequencing (NGS) has become increasingly important in recent years in the field of genomics by sequencing large amounts of DNA or RNA and using bioinformatic analysis for data interpretation [11]. NGS can provide valuable information about genetic alterations, including their impact on the radiosensitivity of tumors with different structures and their response to radiotherapy treatment. It can also reveal cancer gene expression levels and other genomic features that may be associated with tumor radiosensitivity. Although NGS is widely used in oncology, its clinical applications in radiation oncology are still limited. NGS is not currently a common method for predicting tumor radiosensitivity or guiding radiation therapy decisions. The sensitivity of the tumor to radiotherapy is closely related to the proliferation rate. Changes in gene expression due to radiation provide information about the effectiveness of radiotherapy and may change treatment strategies. Furthermore, BCa is commonly treated with high-dose-rate FFF beams in radiotherapy. However, there are no studies based on NGS that investigate the acute radiotherapy response induced by FF and FFF beams for BCa in the literature.

The study established in vivo BCa models in nude mice and treated them with a single dose of 20 Gy radiotherapy at different dose rates. Using NGS technology, the study detected upregulated and downregulated changes in gene expression levels due to FF and FFF beams.

This study aimed to analyze the effect of gene expression level changes on BCa radiotherapy response after irradiation using FF and FFF beams in nude mice models. In addition, the study objectives are to investigate the acute radiobiological mechanisms underlying BCa inhibition.

## Materials and methods

### Establishment of experimental animals and treatment groups

Yeditepe University Animal Experiments Laboratory provided adult 8-week-old female thirty-six outbred athymic nude mice (strain #:007850). The nude mice were kept in standard laboratory and environmental conditions. The humidity, ventilation, and temperature of the rooms were controlled daily using an automated system. The mice were kept in a sterile environment with odorless sawdust as litter. They were housed in HEPA-filtered rooms and IVC cages with their ventilation systems. The cages and litter equipment were sterilized. The mice were given unlimited access to sterile food and were kept in a room with a 12-h light/dark cycle. The study involved nude mice that were randomly divided into five groups. The control group did not receive any treatment, while the BCa group only had breast cancer models created without radiotherapy. The FF-400, FFF-1120, and FFF-1820 radiotherapy groups received a single dose of 20 Gy radiotherapy at different dose rates of 400 MU/min, 1120 MU/min, and 1820 MU/min, respectively (Table 1).

**Table 1:** Experimental study groups

Groups	*n*	Procedures
Group 1 Control	4	*Control group* No action was applied to this randomly selected group
Group 2 BCa	8	*BCa group* BCa models were created in randomly selected outbred athymic nude mice
Group 3 FF-400	8	*FF-400 low dose rate radiotherapy group* BCa models were created and radiotherapy was applied with a single dose of 20 Gy and a low dose rate of 400 MU/min
Group 4 FFF-1120	8	*FFF-1120 high dose rate radiotherapy group* BCa models were created and radiotherapy was applied with a single dose of 20 Gy and a high dose rate of 1120 MU/min
Group 5 FFF-1820	8	*FFF-1820 high dose rate radiotherapy group* BCa models were created and radiotherapy was applied with a single dose of 20 Gy and a high dose rate of 1820 MU/min

*FF* flattening filter, *FFF* flattening filter free, *BCa* breast cancer

### Cell culture and in vivo BCa modeling procedures

The MCF7 cell line belongs to the Luminal A subtype, is estrogen receptor positive (ER+) and shows slower growth with less aggressive behavior. Due to these characteristics, MCF7 provides more controlled experimental conditions**.** In this study, MCF-7 (*ATCC HTB-22*) breast cancer cells for cell culture procedures were provided by the American Type Culture Collection (ATCC). These cells were then incubated in Dulbecco’s Modified Eagle’s Medium High Glucose (DMEM-HG) (*Gibco™*) medium, which contained a final concentration of 2% penicillin/streptomycin (Sigma-Aldrich) and 10% fetal bovine serum (*Sigma-Aldrich*), at 37 °C with 5% CO_2_ for 24 h.

The cells were passaged using trypsin (*Sigma-Aldrich*) when the cell culture dish reached 80% capacity. A total volume of 100 μl containing 1 × 10^7^ cells suspended in a (*1:1 mixture of DMEM-HG and Corning® Matrigel® Matrix (Cat. No. 356255)* was administered subcutaneously to each animal. Tumor growth was monitored by evaluating its progression on specific days following the injection. The mice were euthanized using CO_2_ inhalation after the tumors reached a certain volume. Approximately 10–15 mg of breast cancer tumors were then extracted. 

### Radiotherapy of nude mice

Except for the control (G1) and BCa only (G2) groups, the mice were immobilized with 60 mg/kg ketamine and 8 mg/kg xylazine administered via the ip route during the irradiation process. They were then placed in the supine position on a plexiglass tray under general anesthesia, and radiotherapy was applied. Each mouse received a single dose of 20 Gy, which is equivalent to approximately a conventional biological equivalent dose of 60–70 Gy. The dose was administered using a 6 MV ionizing X-ray Varian Trilogy (*Varian Medical System, Palo Alto, USA*) linear accelerator device at a low dose rate of 400 MU/min (G3) in FF mode and at high dose rates of 1120 MU/min (G4) and 1820 MU/min (G5) in FFF mode. Before radiotherapy, a 10 mm bolus was placed on the radiotherapy field to compensate for dose depth and distribution.

### Euthanasia procedure

The nude mice were anesthetized with 60 mg/kg ketamine and 8 mg/kg ip 48 h after radiotherapy. The mice were euthanized by CO_2_ inhalation, and cancer tissue samples (10–15 mg) were surgically removed and stored under appropriate conditions for molecular investigation of cancer gene expression levels.

### Total RNA extraction from tissue, qualitative and quantitative analysis of RNA

Total RNA from approximately every 10 mg of fresh tumor tissue sample was extracted using the RNAeasy Mini Kit (*Qiagen GmbH, Germany*) according to the manufacturer’s instructions. At the beginning of isolation, tissue samples were thoroughly homogenized for 5 min at 50 Hz using a TissueLyzer LT bead mill and kept on ice for 10 min. Total RNA concentration was performed by fluorometry using the Qubit RNA Broad Range Assay Kit (*Invitrogen, USA*) and the Qubit 4.0 fluorometer (*Invitrogen*). RNA integrity was determined on a TapeStation 4150 using the RNA Screentape Assay (*Agilent Technologies, USA*).

### mRNA library construction, sequencing, and raw data processing

Following isolation, library construction was performed using Illumina Stranded Total RNA Prep, Ligation with Ribo-Zero Plus kit (*Illumina, USA*) according to the manufacturer’s instructions, in an 11 μL volume with 500 ng of total RNA for each sample. Following double-stranded cDNA synthesis, cDNA fragments were converted into libraries by ligating to specific barcoded adapters and amplified using PCR. Library concentration and size were determined by TapeStation 4150 using the D1000 ScreenTape Assay (*Agilent Technologies*). All libraries were pooled, and sequencing of the mRNA libraries was performed on a 2 × 150 bp paired-end NovaSeq 6000 sequencing platform using the S1 Flowcell (*Illumina, USA*).

### RNAseq analysis

Using bcl2fastq2, acquired experimental data is converted to fastq format, and FastQC is used for quality control. HISAT2 is used to align sequence reads to the C57BL_6NJ_v1(GCA_001632555.1) genome assembly and the aligned data are saved in BAM format. Essential step since samtools are used to sort and index the alignment files. Htseq-count is then used to construct count matrices for samples. At this point, count matrices restricted to the protein-coding genes have been generated, and the reference genome assembly and the necessary GFF file for annotation have been downloaded from the Ensembl database. With the DeSeq2 package in R, differential gene expression analysis (DGE) is carried out. Multiple group comparisons (G2 vs. G1, G3 vs. G2, G4 vs. G2, G5 vs. G2) are carried out during the analysis. Results were obtained and filtered according to logFC (abs(logFC) < 1) and adj.p values (= < 0.01). Using the EnhancedVolcano program, the data are finally visualized.

### Gene enrichment and hub gene analysis of differentially expressed genes (DEG)

Using the Cytohub tool in Cytoscape, the hub genes for each treatment group were determined based on MCC. The genes that were identified as hub genes and the genes that they most closely interacted with (neighbor gene interactions) were filtered. The STRING tool was utilized to conduct gene ontology and pathway analysis for the discovered genes, and the Cytoscape program was utilized to show the networks. In addition, the common hub and neighbor genes of each radiation group (G3 vs. G2, G4 vs. G2, G5 vs. G2) were discovered using Venn diagram analysis [12]. With the Genemania tool, PPI analysis and co-expression analysis of the well-known genes were carried out. 

## Results

### Identification of DEG

**Fig. 1:**
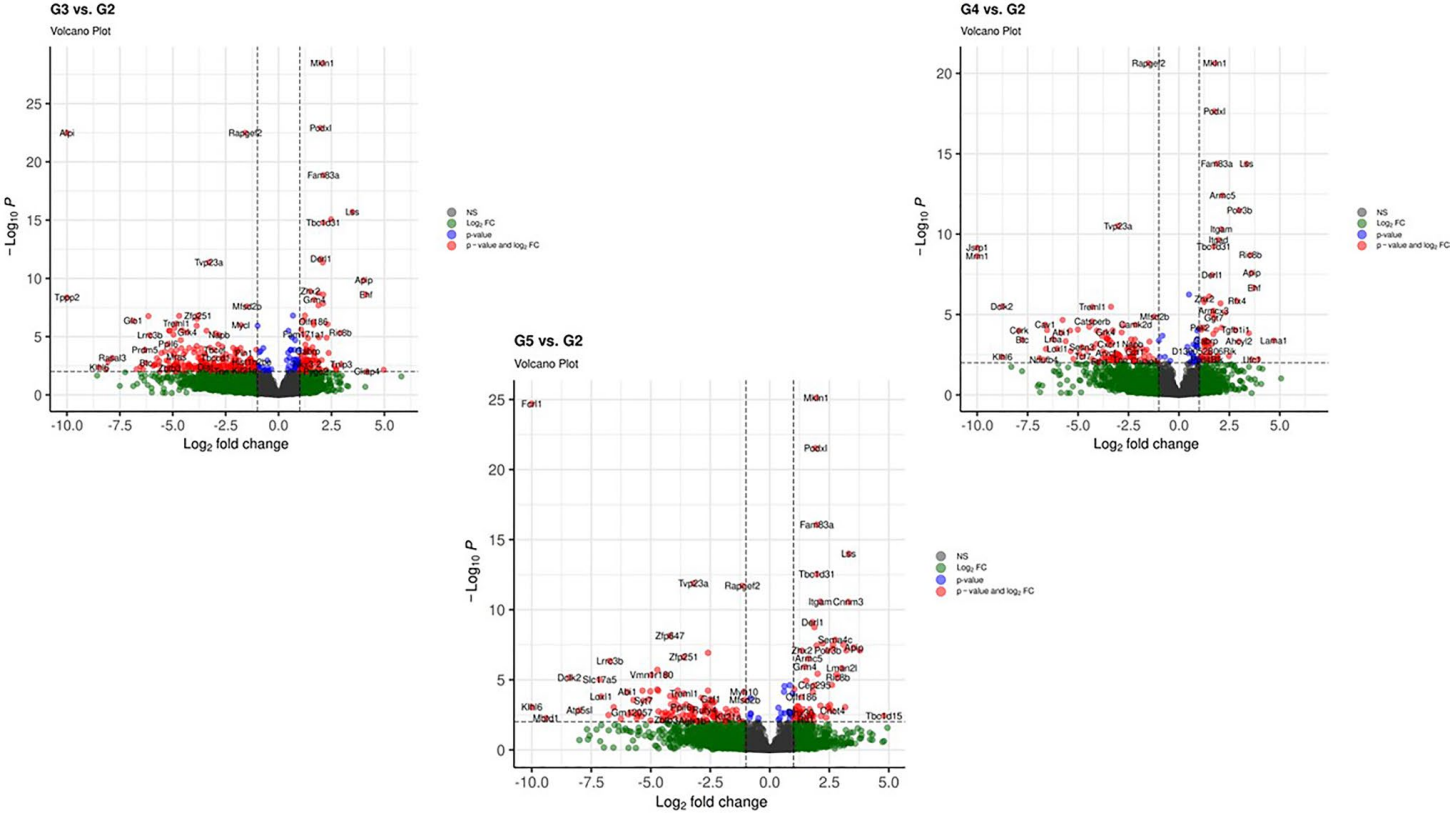
Volcano plot analysis of all radiotherapy treatment groups

According to the DEG analysis results, a total of 1565 genes were found to be significantly altered in the G2 (BCa) vs. G1 (control) groups. When all radiotherapy treatment groups (FF-400, FFF-1120 and FFF-1820) were analyzed, 334 DEGs (231 down, 103 up) in G3 vs. G2 data, 167 DEGs (103 down, 64 up) in G4 vs. G2 data, and 187 DEGs (116 down, 71 up) in G5 vs. G2 data were determined (Supplementary material 1). Their volcano plot analyses are shown in Fig. 1. Some genes that we found to be differentially expressed did not match in pathway analyses. We identified synonyms for these genes using the Mouse Genome Informatics database (MGI) and continued the analysis with these gene names.

### Pathways and analysis of hub genes

**Fig. 2:**
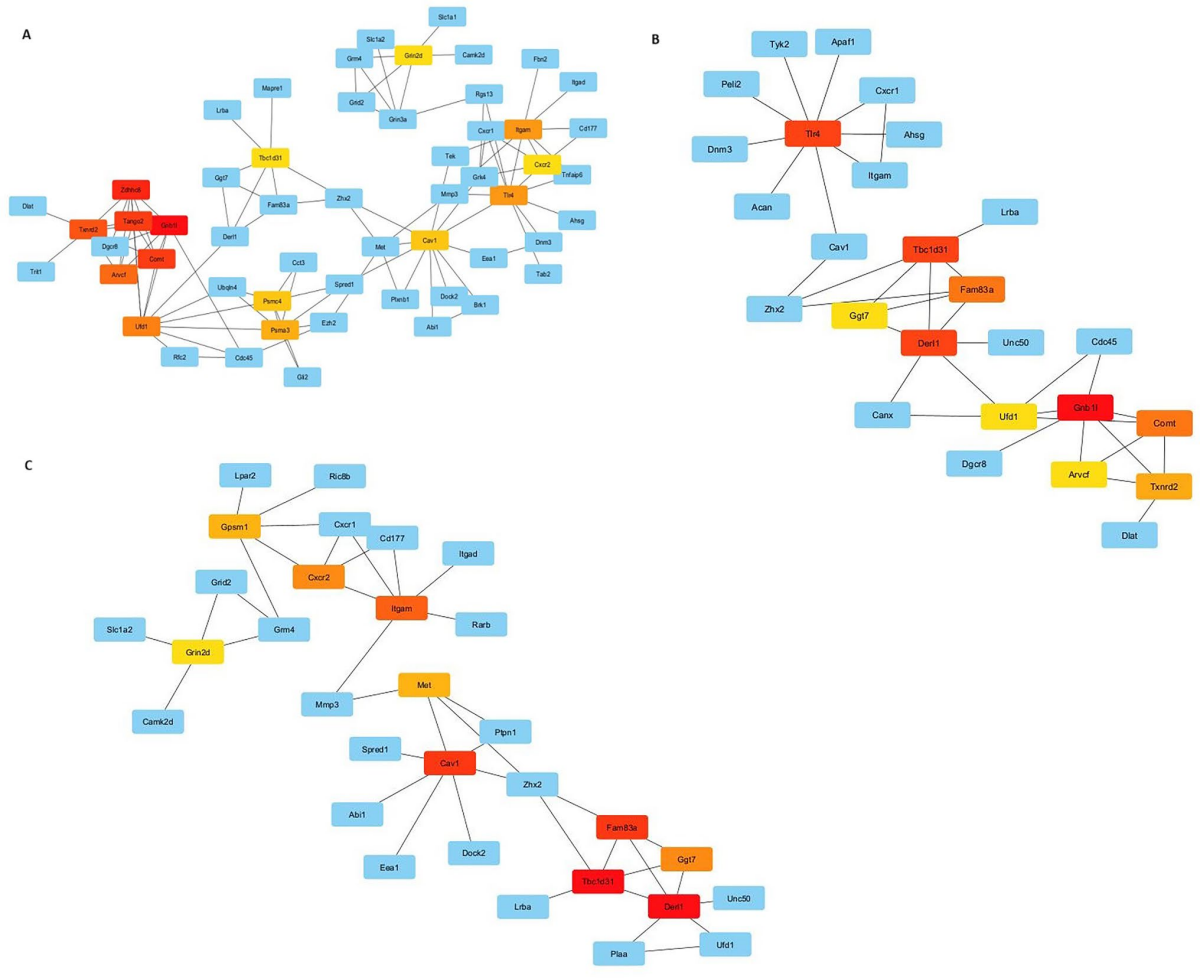
Network analyses of identified hub genes with neighborhood interactions. **A** G3 vs. G2 dataset. **B** G4 vs. G2 dataset. **C** G5 vs. G2 dataset

Hub genes with neighboring genes network analyses were performed separately for each group using Cytoscape (Fig. 2). Pathway (Reactome) and Gene Ontology analyses of the hub genes were also carried out.

For the G3 vs. G2 data set, signal transduction, innate-immune system, and RAF/MAP kinase cascade were found. For the G4 vs. G2 data set, Toll-Like Receptor 4 (TLR4) cascade, innate-immune system, and interleukin-27 signaling, and for the G5 vs. G2 data set, signal transduction and G alpha (i) signaling pathways were detected (Supplementary material 2).

In GO-molecular function analyses for the same groups, protein binding, glutamate receptor activity, identical protein binding, and interleukin-8 receptor activity were prominent for G3 vs. G2 group, while protein binding, interleukin-8 receptor activity, glutamate receptor activity were prominent for G5 vs. G2 group (Supplementary material 2).

In GO- biological process analyses for the same groups, glutamate receptor signaling pathway, regulation of response to stimulus, and regulation of cell communication came to the forefront for the G3 vs. G2 group, while only receptor-mediated endocytosis came to the forefront for G4 vs. G2 group (Supplementary material 2).

Finally, in the GO-cellular component analysis, cell junction, protein-containing complex, receptor complex, and plasma membrane region were detected in the G3 vs. G2 group. In the G5 vs. G2 group, the plasma membrane, plasma membrane raft, and cell periphery came to the fore. Among the other identified biological components in both G4 and G5 groups, synaptic membrane, NMDA selective glutamate receptor complex, and glutamatergic synapse-related mechanisms were determined (Supplementary material 2).

**Fig. 3:**
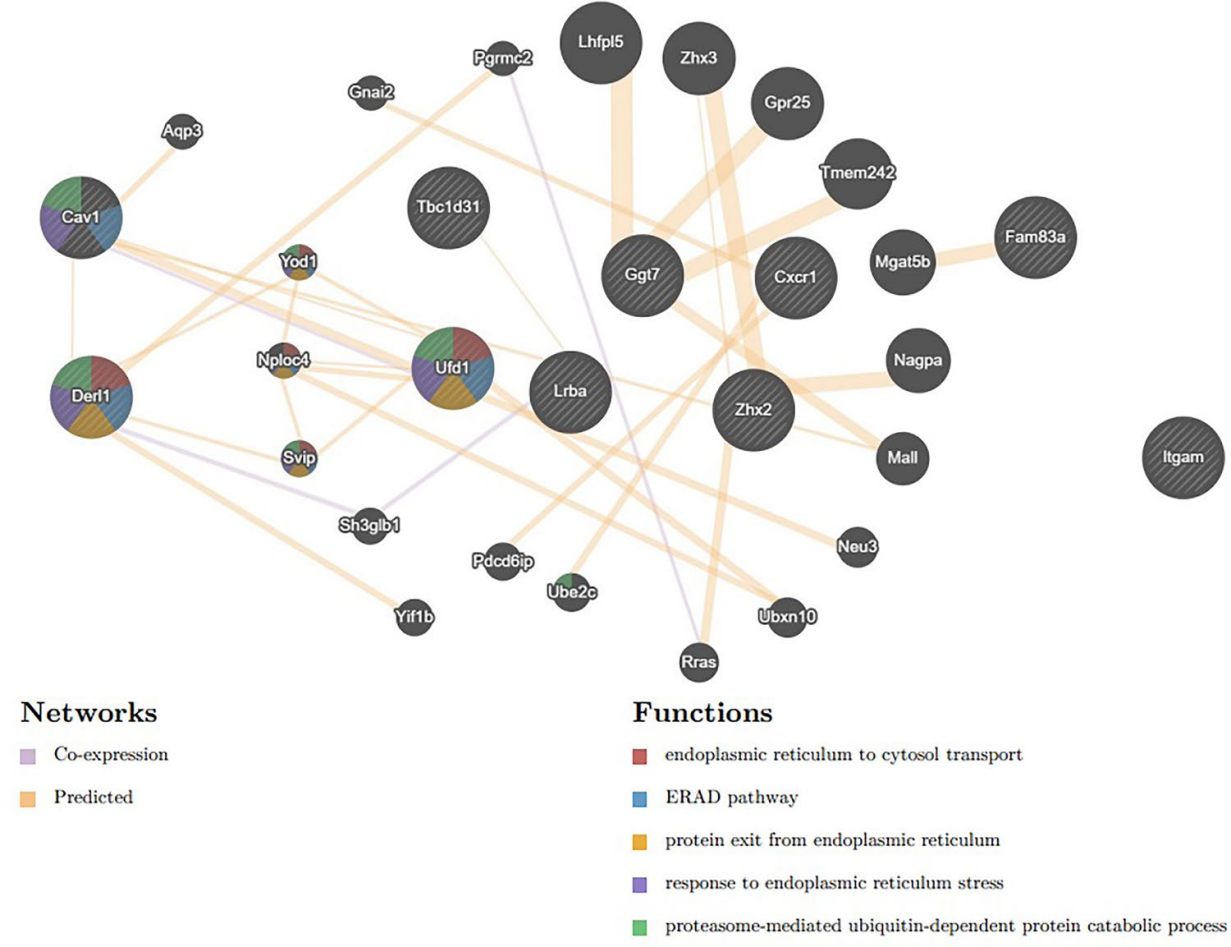
PPI and co-expression analysis of common hub genes of treatment groups

In addition, ten hub (with neighbor interactions) genes were identified as common in the three groups. PPI and co-expression analyses of these genes were performed using the Genemania tool. Among these genes, Derl1, Ufd1, and Cav1 genes were found to be involved in ER-related pathways (Fig. 3).

## Discussion

This study investigated the detection of up regulated and down alterations induced by FF and FFF beams at the gene expression level in breast cancer. The understanding of molecular biology in the treatment process of BCa can lead to a more accurate and effective clinical radiotherapy treatment approach. The resistance of tumors such as BCa to radiotherapy due to genetic mutations or changes in the tumor microenvironment may limit treatment success and lead to disease progression [13]. The literature has not yet clarified the genetic mechanisms, molecular events, and markers underlying radiotherapy response for BCa [14]. Therefore, it is important to identify the upregulation and downregulation of genes during breast cancer radiotherapy with FF and FFF beams [15].

Although FFF beams are considered dosimetrically superior, there is limited radiobiological information in the literature regarding in vitro and in vivo cancer studies. Therefore, it is crucial to determine the precise genetic outcomes following SRS and SBRT treatment using FFF beams. There are many in vitro studies comparing the radiobiological effects of FF and FFF radiation on the survival of different cancer cell lines [16–20]. However, there is limited knowledge regarding the potential effects of FFF beams on cancer cells. Previous in vitro radiobiological studies have shown conflicting results [21, 22]. The in vivo radiobiological consequences of the FFF clinical dose rate effect are still uncertain [23, 24]. Thus, it is important to investigate whether there is a radiobiological difference between FF and FFF beams in terms of breast cancer gene expression levels.

Radiotherapy response in cancer depends on various treatment options and tumor molecular characteristics. NGS can be used to predict radiation response in cancer. Genetic variants and biological mechanisms play a crucial role in determining radiotherapy responses. Therefore, it is essential to consider multiple genes and their interactions to predict tumor radiation response accurately. Predicting tumor radiation response based on NGS is challenging due to the complexity of the underlying biological mechanisms. The existing literature on these mechanisms is limited, making it difficult to predict radiation response accurately using NGS. The study evaluated the effects of radiotherapy on molecular mechanisms and alterations in tumor structure using different dose rates (FF vs FFF). It investigated the detection of upregulated and downregulated changes in gene expression levels caused by FF and FFF beams in BCa.

According to the molecular function analysis performed in this study, glutamate receptor activation was prominent in both G3 and G5 groups. The coordinated exchange of biochemicals and energy within and between cell components is made possible by intricate networks known as metabolic networks. Within this milieu, a select few molecules among them, glutamate play important pivotal roles in crucial pathways that need the synchronized interactions of several intermediaries. Reprogrammed metabolism enables the rapid proliferation of cancer cells. Biochemical alterations of cancer metabolism are made possible by two major mechanisms: glutamine catabolism and glycolysis.

There are both neuronal and non-neural cell components in the glutamatergic system. Recent studies have shown that membrane glutamate transporters and ionotropic and metabotropic glutamate receptors regulate a wide range of processes involved in the pathophysiology of non-neurological diseases, particularly those related to inflammatory and oncological conditions. These results demonstrate these receptors’ potential value as therapeutic targets or biomarkers. Ionotropic glutamate receptors are glutamate-gated ion channels and are classified as NMDA (N-methyl-d-aspartate) [25]. The Grin2d gene, a subunit of this class, is highly expressed in many non-neuron cancer types such as colorectal [26], pancreas [27], and lung [28]. In our study, this gene was found to have low expression in all treated groups (G3, G4, and G5) compared to the untreated cancer group (G2). In a study conducted in triple-negative breast cancer (MDA-MB-231) cells, the effect of miR-129-1-3p targeting the highly expressed Grin2d gene was evaluated and it was found that this miRNA caused suppression of important mechanisms such as cell growth and migration by suppressing Grin2d [29]. Therefore, the fact that the effect of radiotherapy reacted in the same direction in our study suggests that especially the treatment methods applied have potential effects to stop the vital functions of cancer cells by suppressing Grin2d, which is involved in calcium metabolism.

The biological role of CaMK2 in breast cancer progression has been the subject of recent studies. One study showed that inhibition of CaMK2 signaling partially abolished ANO1’s promotion of cell viability and proliferation in ANO1-replicated and overexpressed breast cancer and concluded that CaMK2 may play a critical role in cellular proliferation and oncogenesis in breast cancer [30]. In another study, expression and phosphorylation of CaMK2α at T286 were significantly elevated in breast cancer specimens and lymph node metastasis tissues, and pharmacological inhibition of CaMK2α using both AIP and KN-93 attenuated migration and invasion of MDA-MB-231 cells [31]. In a study conducted with ovarian cancer, it was shown that high expression of the CAMK2D gene conferred resistance to cells in Cisplatin treatment [32]. In our study, the expression of the CAMK2D gene decreased as the radiotherapy dose rate increased. This suggests that CAMK2D may negatively affect cancer formation mechanisms by becoming less expressed.

In the co-expression analysis of the common hub genes determined between the groups, especially the ERAD (endoplasmic reticulum–associated protein degradation) which is a specific cellular pathway that targets misfolded proteins of the endoplasmic reticulum for ubiquitination and subsequent degradation by a protein-degrading complex was found to be significant. Among the prominent genes in the ERAD pathway, Ufd1 and Cav1 genes were found to be highly expressed in the cancer group (G2) compared to the (G1) healthy control group (G2 vs. G1), while they were low expressed in all treatment groups (G3, 4 and 5). An intrinsic membrane protein known as caveolin-1 (Cav-1) can take part in signal transduction and tumor progression, two processes that are related to the proliferation of tumor cells [33]. Cav-1 exhibits anti-cancer [34] as well as cancer-promoting properties. In breast cancer, Cav-1 expression can range from high to low, yet observations in the literature are contradictory. Consequently, opinions about whether Cav-1 is an oncogenic gene or a tumor suppressor in breast cancer are divided. The function of nuclear protein localization-4 and valosin-containing protein is to form a complex with ubiquitin recognition factor in ER-associated degradation 1 (Ufd1), which is required for the degradation of ubiquitinated proteins. This complex is also in charge of the breakdown of the mitotic spindle and the creation of a closed nuclear envelope following mitosis. In a study on breast cancer, Wang et al. found that by directly binding to the 3′UTRs of UFD1 and NT5E, increased expression of PCBP2 positively controls their subsequent expression and promotes breast cancer progression [35]. In a study with lymphoblastic leukemia, they showed that MYC overexpression enhances the UPR system and simultaneously the ERAD system through transcriptional upregulation of UFD1. They also reported that inhibition of Ufd1 contributes to the apoptotic process [36].

This study demonstrates that while there are some variations in gene expression levels such as Camk2d, between FF and FFF beams, there is no significant difference. However, the prominence of similar molecular mechanisms, such as the ERAD pathway and glutamate receptor activation, with the use of FFF beams suggests that high-dose-rate radiotherapy plays an active role, especially in these pathways.

In this study, the effects of FF and FFF radiotherapy beams on gene expression levels in the acute phase (after 48 h) were investigated. However, molecular responses in the subacute and chronic phases were excluded from the evaluation. Considering that genetic responses to radiotherapy may change over time, further studies including time points that will reveal long-term transcriptomic regulations are required. This is a limitation of this study and it is recommended that genetic responses changing over time should be analyzed in future studies. In addition, only in vivo model was used in the present study and the gene expression changes obtained were not confirmed at the cell culture level. This is a limitation and it is planned to support the functional effects of the identified key genes (e.g., Ufd1, Cav1, Grin2d) with in vitro studies in the future. It is also planned to include subtypes such as triple-negative and HER2+ in future studies. Another limitation of this study is that only the dose rate effect was analyzed using a fixed dose (20 Gy). However, the addition of different dose values and long-term follow-up is important to evaluate the dose–response relationship more comprehensively. Therefore, analyses of multiple dose protocols and long-term effects of radiotherapy are planned in other studies.

## Conclusion

Determining the expression changes in BCa at different dose rates of FF and FFF beams is an important aim of this study. It is concluded that the FFF beam significantly alters the processes underlying the ERAD pathway and glutamate receptor activation compared to the FF beam. These mechanisms need to be further elucidated by in-depth investigations. Considering that genetic responses to radiotherapy may change over time, further studies are needed to reveal the transcriptomic regulations of multiple dose protocols and long-term effects of radiotherapy. 

## Supplementary Information

Below is the link to the electronic supplementary material.

Supplementary file1 (XLSX 236 KB)

Supplementary file2 (XLSX 24 KB)

## Data Availability

Not applicable.

## Abbreviations

FF: Flattening Filter
FFF: Flattening Filter Free
BCa: Breast cancer
DEG: Differentially expressed genes
SBRT: Stereotactic Body Radiation Therapy
SRS: Stereotactic Radiosurgery
VMAT: Volume-Modulated Arc Therapy
NGS: Next Generation Sequencing
ATCC: American Type Culture Collection
NMDA: N-Methyl-d-aspartate
